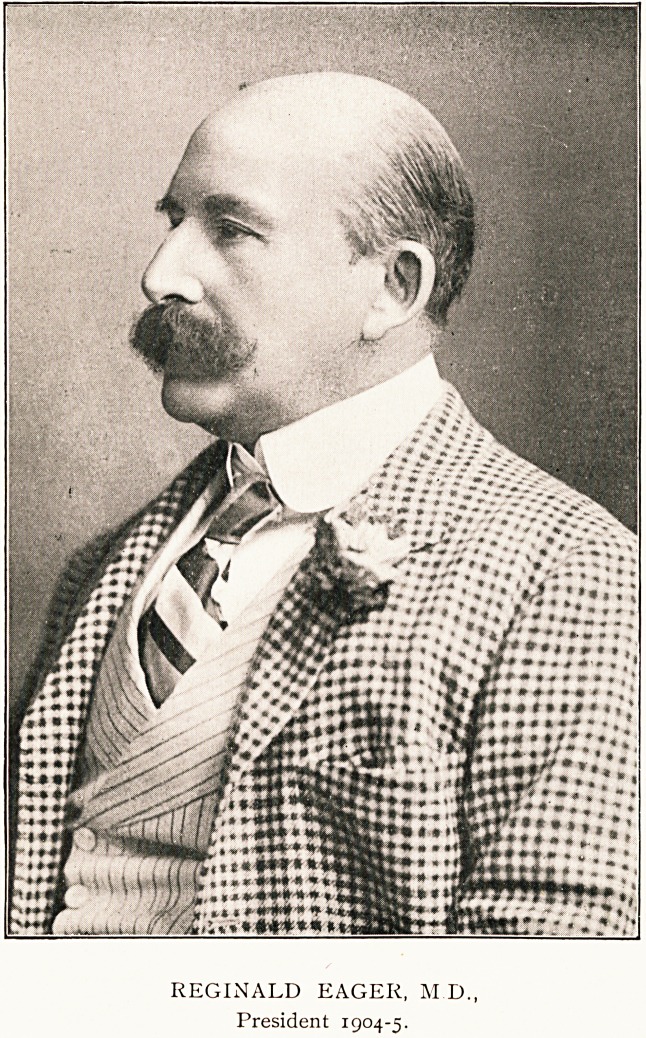# Reginald Eager

**Published:** 1915-12

**Authors:** 


REGINALD EAGER, M.D.,
President 1904-5.

Lieut.-Col. MICHELL CLARKE read a paper on **Gun-Shot Injuries of Nerves.** The PRESIDENT and Messrs. MOORE and LANSDOWN discussed the paper.

Major LANSDOWN read a paper on **The Localisation of Bullets.** (See p. 157.)

Capt. STACK read a paper on **Gun-Shot Wounds of the Eye** (See p. 198.)

### Obituary.

### REGINALD EAGER, M.D., M.R.C.S., L.S.A., M.B.

DR. REGINALD EAGER was born at Guildford, Surrey, in April, 1841. His career as a student at Guy's Hospital was a successful one. He took the M.R.C.S. in 1866, L.S.A. in 1867, M.B. (Lond.) in 1868 (taking the Gold Medal in Forensic Medicine and 1st Class Honours in Medicine and Midwifery), and M.D. (Lond.) in 1869. His paper "On Sleep" was awarded the Guy's Hospital Physical Society's Prize as the best paper read before them during that year. Later he was President of this Society. He held the appointments of House Surgeon and Obstetric Resident at Guy's Hospital. After leaving Guy's he was for five years Medical Superintendent of St. Luke's Hospital for Mental Diseases.

In the spring of 1875 he and the late Mr. T. G. Seymour bought Northwoods House, where he resided until he retired in 1913.

In 1910 he first diagnosed symptoms of the malady which was the cause of his health breaking up. For a little over five years he bore his illness with uncomplaining fortitude and courage, doing work and getting about in a way that caused astonishment to those around him.

Dr. Eager was for many years (1879 to 1913) Lecturer on Medical Jurisprudence at the Bristol Medical School. At one time he was a member of the Gloucestershire County Council and Education Committee. He was a member of the Bristol Diocesan Conference, Vice-President for many years of the Bristol District Union of the English Church Union, member of the Bristol Church Day Schools' Association, Churchwarden of Frampton Cotterell Church for twenty-five years, and Chairman and Correspondent of the Church of England Schools. He was a well-known authority on Church matters, and a member of St. Paul's Ecclesiological Society, London.

He had travelled widely, and had visited many places very much out of the ordinary routes. His reminiscences of these trips were interesting and illuminating. He was an artist of no mean order. He was a keen gardener, and during his later years grew and was much interested in the cultivation of orchids.

In private life he was genial and affectionate, hospitable and liked to have people around him.

## Figures and Tables

**Figure f1:**